# The necessity of prophylactic central lymph node dissection in clinically n0 papillary thyroid carcinoma: perspective from the endemic region

**DOI:** 10.1007/s00423-025-03667-y

**Published:** 2025-03-28

**Authors:** Tugba Matlim Ozel, Yigit Soytas, Sezer Akbulut, Aykut Celik, Gorkem Yildiz, Huseyin Karatay, Serkan Sari

**Affiliations:** 1 Department of General Surgery, Division of Endocrine Surgery, University of Health Sciences Turkey, Basaksehir Cam and Sakura Training and Research Hospital, Istanbul, Turkey; 2 Department of Pathology, University of Health Sciences Turkey, Basaksehir Cam and Sakura Training and Research Hospital, Istanbul, Turkey

**Keywords:** Papillary thyroid carcinoma, Prophylactic central lymph node dissection, TNM staging, Safety, Lymph node metastasis

## Abstract

**Background:**

Prophylactic central lymph node dissection (pCND) in papillary thyroid carcinoma (PTC) is still a matter of debate. Therefore, we aimed to identify the factors affecting central lymph node metastasis (CLNM) in patients with clinically node-negative (cN0) PTC.

**Methods:**

This retrospective study included 248 patients with cN0 PTC who underwent total thyroidectomy (TT) or TT + pCND. Clinicopathological associations among CLNM, complication rates and the effect of pCND on staging were assessed. Risk factors (RFs) and the pattern of lymph node metastasis (LNM) in PTC patients were studied via multivariate analysis.

**Results:**

A total of 216 patients underwent pCND, and *58.8%* (127/216) had positive CLNM. Male patients, aged < 41 years, and those with lymphatic invasion were identified as RFs for CLNM, with odds ratios of 2.59, 2.26, and 4.09, respectively. Among the 216 patients, 65 (30%) had transient hypoparathyroidism (HPT), and 20 (9.3%) had permanent HPT. Transient recurrent laryngeal nerve (RLN) palsy occurred in 15 (6.9%) patients, and permanent RLN palsy occurred in 3 (1.4%) patients. Over 55 years of age, 46.7% of patients were upstaged according to the American Joint Committee on Cancer (AJCC) TNM staging system, and 14.2% (*n* = 18) of the 127 patients with CLNM were upgraded according to the American Thyroid Association (ATA) risk stratification system *(RSS)*.

**Conclusion:**

Taken together, in terms of the high incidence rate of CLNM in cN0 PTC patients; We believe that routine pCND, which can be performed with low morbidity rates, is optimal for cN0 PTC patients during their first treatment, especially for those with RFs for CLNM.

**Clinical trials number:**

NCT05873283.

## Introduction

Thyroid carcinoma (TC), the most common endocrine malignancy, has been diagnosed worldwide with increasing frequency in recent decades, especially among women [1–3]. PTC is the most common thyroid malignancy, accounting for approximately 90% of new cases of TC in iodine-sufficient areas of the world [4]. PTC has become the second most prevalent carcinoma among women and eighth among men in Turkey [5]. According to the 2023 cancer statistics data of the Ministry of Health, the prevalence of TC in women aged 25–49 in Turkey ranks 2nd after breast cancer. Turkey is among the countries with a high incidence rate such as North America, China, Australia, Italy, Germany, Ukraine, with an incidence rate of 7.8% per hundred thousand people in terms of TC. Thyroid cancer in Turkey has the highest incidence among the European region, upper-income countries, and middle-income countries [5].

The overall incidence of PTC is increasing; however, most PTC patients have excellent prognoses [6]. Despite this favorable outcome, PTC has an average incidence of 60% of cervical LNM, which most commonly occur in the central neck compartment [7, 8]. Clinically detectable macrometastatic lymph nodes (LNs) are found in up to 35% of patients; 80% of metastases present with microscopic LNs, resulting in a high locoregional recurrence (LRR) rate in the paratracheal region, which has been reported to be up to 30% [7, 9]. Currently, there are no non or minimally invasive methods that are completely reliable for detecting all of the metastases. As many as half of the nodes found later in surgery can be missed during the initial ultrasound (US) examination [10].

There is no controversy about the operation of therapeutic central neck dissection (CND) when clinical LNMs exist in the central neck compartment, while pCND in clinically negative (cN0) PTCs remains controversial [11, 12]. According to previous studies, the advantages of routine CND include a reduction in the LRR, a reduction in the risk for complications associated with reoperation at level VI, and a higher rate of patients with low serum levels of thyroglobulin (Tg) and antithyroglobulin antibodies (Anti-Tg), which facilitate tracking and potentially improve disease-free survival (DFS) [13–15]. In addition, pCND could also help in more precise staging of PTC, since nodal positivity could convert patients from cN0 to pN1a, leading many patients over 55 years of age from AJCC stage I to AJCC stage II, which in turn changes the therapeutic choices [16, 17]. However, other studies have demonstrated that occult LNM increase the LRR but do not affect DFS and that therapeutic CND in the event of central compartment recurrence can be performed with low morbidity, which is no greater than an initial pCND [18, 19]. Considering that the risk of postoperative complications such as RLN injury and HPT following pCND outweighs the potential benefits, the routine use of pCND in PTC is not recommended [20, 21].

The recommendations of guidelines in different countries and regions are not the same. The 2015 ATA Guidelines and the 2019 European Society for Medical Oncology (ESMO) Clinical Practice Guidelines recommend pCND + TT only in advanced primary tumors or in the presence of certain RFs and lateral neck node involvement [22, 23]. The 2020 Japan Associations of Endocrine Surgeons Clinical Practice Guidelines suggest that primary tumor surgery with pCND to T1B and above tumors is recommended with risk assessment [24].

The mortality rate of PTC patients is low and stable, and in the last decade, there has been a paradigm shift toward less aggressive surgical treatment of PTC. The main challenge still remains to identify patients at greater risk of recurrence who require more extensive surgical and postoperative radioactive iodine (RAI) treatment but also importantly, to avoid overtreatment of lower-risk patients. The aim of this study was to examine the incidence and RFs of CLNM in patients with cN0 PTC and the morbidity in patients with PTC treated with TT + bilateral/ipsilateral CND compared with those in patients who underwent TT alone. The influence of CLNM identified in the implementation of pCND, on the migration of AJCC TNM staging and ATA RSS in patients with cN0 PTC was also evaluated in our study. The data from this study may provide additional guidance for surgical and therapeutic decisions for cN0 PTC patients from endemic regions.

## Materials and methods

### Study design

We conducted a prospectively planned study in which the data were collected and analyzed retrospectively. The study was conducted at the Department of General Surgery University of Health Sciences, Basaksehir Cam and Sakura Health Practices and Research Center. The study was approved by the local ethics committee of the Basaksehir Cam and Sakura Health Practices and Research Center (EK- 2021.03.10).

We performed a study between July 2020 and September 2023. A total of 367 patients underwent surgery for Bethesda 5 (B5) and Bethesda 6 (B6) thyroid nodules [25]. Patients whose data are summarized in Table 1 were excluded. Among these 87 B5 and 174 B6 patients who had cN0 disease and who underwent surgery were included in the study. Among the 261 patients, 78 B5 nodules (90%) and 170 B6 nodules (98%) had histologically proven PTCs. Bilateral pCND was performed in patients who were young, who has a family history of thyroid cancer and a history of radiation therapy, nodules larger than 4 cm, when thyroid cancer was bilateral, when tumors were located at isthmus and when tumors found to be extrathyroidal during surgery, regardless of tumor diameter. Total thyroidectomy was performed in patients who were elderly, had high morbidity due to additional diseases, had tumors smaller than 1 cm and located intrathyroidal, and in cases where there was loss of signal or adverse EMG event observed during surgery. Finally, 248 patients who underwent TT or TT + unilateral/bilateral pCND with cN0 histologically proven PTC were analyzed.

**Table 1:** Inclusion and exclusion criteria

Inclusion criteria	Exclusion criteria
Age > 18 years B5 and B6 nodules confirmed by fine needle aspiration (FNA) cytology (*n* = 367) No evidence of lymphadenopathy on clinical examination No evidence of lymphadenopathy on preoperative US	Subtotal or hemithyroidectomy as definitive procedure (*n* = 19) Evidence of nodal disease on preoperative US or clinical examination (*n* = 18) Benign final histology (*n* = 13) Lateral neck dissection performed at initial surgery (*n* = 62) Distant metastatic disease (*n* = 3) Patients with prior thyroid surgery (*n* = 4)

First, the study group comprised patients who underwent TT without pCND (Group A). These patients were compared with patients who had TT + pCND (Group B). The Group B patients were subsequently divided into two groups on the basis of pathological analysis: those without CLNM (Group pN0) and those with CLNM (Group pN1).

The following parameters were prospectively registered in a specifically designed database (Microsoft Excel, Microsoft Corporation, Redmond, WA): patient demographics; preoperative data; surgical procedures; postoperative complications, including RLN palsy, HPT, neck hematoma and wound infection; histologic subtype; tumor bilaterality; multifocality; extrathyroidal extension (ETE); lymphovascular invasion; tumor size; central LN status; T stage; number of removed and metastatic LNs; TNM staging; and the results of follow-up evaluations. Staging of PTCs was performed via the eighth edition of the AJCC staging system. Risk categories were defined on the basis of the 2015 ATA guidelines [22]. A comparative analysis among the groups concerning the registered parameters was performed. All patients provided written informed consent for the study.

### Preoperative assessment

All the patients included in the study had undergone a preoperative physical examination, neck US, FNA of suspicious nodules, and measurement of serum thyroid hormones, thyrotropin (TSH), Tg, and anti-Tg; preoperative fiber nasopharyngolaryngoscopy was also routinely performed for vocal cord functions. The indication for surgery is based on suspicious nodules that demonstrate US characteristics typical for malignancy, are cold in thyroid scintigraphy and in which FNA determines B5 / B6. All US examinations and US-guided FNAs were performed in the Department of Radiology by a single experienced radiologist trained in neck US. Every patient underwent an US examination of the thyroid gland, the central and lateral LNs. In addition, US-guided FNA of the “leading” thyroid nodule was conducted. Cytological and histopathological examinations were performed at the clinical institute of Pathology.

### Surgical technique

All surgical procedures were performed by experienced endocrine surgeons in our hospital (each having more than 100 thyroid operations per year) who followed a standard technique described by Barczynski et al. [26]. Surgery was started from the thyroid lobe where the tumor was located. Contralateral lobectomy was completed in cases without loss of signal (LOS)/ adverseEMG changes. CND was performed after thyroidectomy if no problems developed in the EMG data. Surgical procedures were accompanied by extended dissection of the RLN via intermittent intraoperative neuromonitoring. When feasible, all four parathyroid glands were visualized and carefully dissected from the thyroid capsule. If the blood supply of a parathyroid gland could not be maintained, the gland was removed and then immediately autotransplanted into the sternocleidomastoid muscle on the side opposite the tumor.

### Definitions

#### Postoperative disturbance of RLN function

All operations were performed with intermittent or continuous intraoperative neural monitoring (IONM). The equipment setup, anesthesia, standards of IONM, EMG definitions, and data interpretation of IONM were applied in accordance with the International Intraoperative Neural Monitoring Study Group (INMSG) guideline [27]. During the operations, adverse EMG parameters were defined as an amplitude decrease of 50% or more of the baseline value, or a latency increase of 10% or more. Recovery was defined as amplitude recovery to > 50% of the initial baseline amplitude. LOS was defined as an amplitude of < 100 µV [27]. In the case of LOS or if no improvement was achieved in adverse EMG changes with negative glottis response, surgery was aborted considering staged surgery and postoperative fiber nasopharyngolaryngoscopy was performed. Temporary RLN palsy was defined as decreased or absent vocal cord mobility resolving within 6 months of surgery. Permanent RLN palsy was defined as vocal cord dysfunction persisting beyond 6 months after initial surgery.

#### Postoperative HPT

Serum calcium levels and PTH levels were assayed on the first postoperative day and then on the basis of the evolution of clinical and biochemical parameters. Medication was started prophylactically, and no patient was allowed to develop symptoms of hypocalcemia. Calcium replacement (1.5–3.0 g calcium daily) was administered when the total serum calcium level was < 8 mg/dL, and calcium plus calcitriol (0.25–1.0 µg/day) was given when PTH was < 10 pg/ml (reference range: 15–65 pg/ml). A serum calcium level < 8 mg/dL (reference range: 8.6–10 mg/dl) with a subnormal serum PTH level (< 10 pg/ml) was defined as transient HPT if the level returned to a normal value within 12 months after the withdrawal of calcium therapy. Hypocalcemia with a serum PTH level < 10 ng/l for more than 12 months after surgery, requiring substitution with calcium with or without calcitriol, was regarded as permanent HPT [28].

### Postoperative follow-up

Radioactive iodine remnant ablation was performed with iodine-131 (I131) after surgery, followed by thyroxine withdrawal in selected patients. Suppressive L-thyroxine therapy was routinely administered with the thyroxine dose adjusted to a risk TSH level below 0.1 uIU/ml for high-risk patients and 0.1–0.3 µIU/ml for low-risk patients. Routine follow-up (every 3 months in the first year and then yearly) included neck US, estimation of serum TSH and basal Tg levels, and measurement of anti-Tg. A basal serum Tg level < 0,2 ng/ml in any patient on levothyroxine suppressive therapy or TSH-stimulated Tg < 1 ng/ml was considered to indicate a low risk of recurrent disease [22].

### Statistical analyses

The normality assumption of continuous variables was tested with the Kolmogorov‒Smirnov test. Categorical variables are presented as frequencies (n, %); continuous variables are presented as the means and standard deviations. Comparisons between two groups in continuous variables were performed with the Mann‒Whitney U test. Comparisons between more than two groups were made with the Kruskal‒Wallis H test. Pearson’s chi-square test and Fisher’s exact test were used to compare qualitative data. Multivariate logistic regression analysis was used to determine the independent variables associated with the dependent variables. Receiver operating characteristic (ROC) curve analysis and the Youden index were used to determine the most appropriate age group for pathologic LN involvement. The results were evaluated at the 95% confidence interval, and significance was evaluated at *p* < 0.05. All the statistical calculations were performed with SPSS software version 26 (IBM Corp., Armonk, NY, USA).

## Results

### Comparison of TT vs. TT + pCND

The study included 198 women and 50 men with a mean age at diagnosis of 46.16 ± 12.49 years. Group A included 32 patients who underwent TT only, whereas Group B included 216 patients who underwent TT + pCND. Data were analyzed on the basis of the classification of patients into groups A and B. The two groups were similar in terms of sex distribution, pathological tumor size, and pathological prognostic features, such as the incidence of tumor multifocality, ETE, capsular invasion, and vascular invasion. In the TT group, 24 patients had no tumor at the surgical margin and 7 patients had tumor adjacent to the surgical margin and 1 patient had multifocal tumors with negative and adjacent to the surgical margins. In the TT + pCND group, 176 patients had negative surgical margins, 33 patients had tumors adjacent to the surgical margin, 5 patients had multifocal tumors with negative and adjacent to the surgical margins, and in 2 patients tumor showed continuity at the surgical margin in the focal area. However, the presence of aggressive histological subtypes, lymphatic invasion and multifocal tumor frequency were quite high in both groups. When the two groups were evaluated together, these rates were 31.8%, 74.6%, and 68%, respectively. The Group B patients with pCND were significantly younger (mean ± SD, 45.2 ± 12.1 vs. 52.8 ± 13.4; *p* = 0.004) than the Group A patients were and had a higher rate of lymphatic invasion noted in the primary tumor (77% vs. 59%; *p* = 0.034) Patient demographics and clinicopathologic characteristics are summarized in Table 2.

**Table 2:** Demographic and clinicopathologic characteristics by surgical type

Variables	All (*n* = 248)	Group A (*n* = 32)	Group B (*n* = 216)	*P* value
**Age (year)**, ***mean ± SD***	46.16 ± 12.49	52.84 ± 13.39	45.17 ± 12.07	**0.004** ^**a**^ *
**Sex**, ***n(%)***				0.094^b^
Female	198(79.8)	22(68.8)	176(81.5)	
Male	50(20.2)	10(31.3)	40(18.5)	
**pT Stage**, ***n(%)***				0.086^c^
T1A (< 9 mm)	108(43.5)	11(34.4)	97(44.9)	
T1B (10–19 mm)	93(37.5)	10(31.3)	83(38.4)	
T2 (20–39 mm)	34(13.7)	7(21.9)	27(12.5)	
T3 (≥ 40 mm or ETE regardless of tumor size)	13(5.2)	4(12.5)	9(4.2)	
**Histologic subtype**, ***n(%)***				
Classic + Mixt type	141(56.9)	16(50)	125(59.7)	0.165^b^
Follicular	28(11.3)	4(12.5)	24(11.1)	
Tall cell/Hobnail	79 (31.8)	12(37.5)	67(29.2)	
**Multifocality**	169(68.1)	18(56.3)	151(69.9)	0.122^b^
**Capsular invasion**	89(35.8)	15(46.9)	74(34.3)	0.381^b^
**Extrathyroidal extension**	20(8)	3(9.4)	17(7.6)	0.471^b^
**Vascular invasion**	29(11.7)	5(15.6)	24(11.1)	0.509^b^
**Lymphatic invasion**, ***n(%)***	185(74.6)	19(59.4)	166(76.9)	**0.034** ^**b**^ *
**LNs removed**, ***n(%)***	217(87.5)	5(15.6)	216(100)	N/A
**Number of LNs removed**, ***mean ± SD***	14.48 ± 8.91	2.00 ± N/A	14.54 ± 8.89	N/A
**Lymph node metastasis**,** n(%)**	128(59)	1(3.1)	127(58.8)	N/A
**Number of metastatic LNs**, ***mean ± SD***	3.26 ± 2.88	1.00 ± N/A	3.28 ± 2.88	N/A

*p < 0.05, a: Mann‒Whitney U test, b: Pearson chi‒square test, c: Fisher’s exact test, SD: standard deviation, N/A: not available

As expected, group B patients had a significantly greater number of LNs harvested during the original operation. In group B, 127 patients (58.8%) had nodal metastatic disease proven at final histology. The mean number of LN removed after pCND was 14.54 ± 8.89. Ipsilateral pCND was performed in 168 (78%) patients, and 48 (22%) had bilateral pCND. In group A, despite no formal CND, 5 patients had perithyroidal LNs found during pathological examination of the thyroid. One (3.1%) of these patients had LNM. RAI therapy was used in 199 (80%) of the 248 patients: 23 patients (71.9%) who had TT versus 176 (81.5%) who had TT + pCND (*p* = 0.203). The RAI dose in the TT + pCND patient group were higher (mean ± SD, 110.87 ± 39.04 vs. 130.89 ± 30.19; *p* = 0.001).

In the study population as a whole, 27 (10.8%) patients and over 55 years of age 46.7% of patients were upstaged according to the AJCC staging system for PTC. In group A, 1 patient (3.12%) was upstaged to stage II. In group B, 26 patients (12%) were upstaged to stage II on the basis of LN involvement.

The operative complications are summarized in Table 3. Transient HPT was observed in 71 (28.6%) patients, and transient RLN palsy was observed in 23 (9.3%) patients. Permanent HPT was observed in 24 (9.7%) patients, and permanent RLN palsy was observed in 4 (1.6%) patients. However, there was no statistically significant difference in the incidence of transient-permanent HPT and permanent RLN palsy between groups A and B (*p* > 0.05). The rates of temporary RLN palsy were greater in Group A (25% vs. 6.9%; *p* = 0.004). Another notable finding is that only 2 of the cases of RLN injury occurred during pCND, and 1 of them became permanent.

Autografting of parathyroid glands was more common in Group B than in Group A, but this difference was not statistically significant (12.5% versus 3.1%, *P* = 0.143). The rates of wound infection and postoperative hemorrhage were equivalent between the 2 groups.

**Table 3:** Operative complications by surgical type

Variables	All (*n* = 248)	Group A (*n* = 32)	Group B (*n* = 216)	*P* value
**Hypoparathyroidism**, ***n***(%)				
Transient	71(28.6)	6(18.8)	65(30.1)	0.185^a^
Permanent	24(9.7)	4(12.5)	20(9.3)	0.527^b^
**RLN palsy**, ***n(%)***				
Temporary	23(9.3)	8(25)	15(6.9)	**0.004** ^**b**^ *
Permanent	4(1.6)	1(3.1)	3(1.4)	0.427
**Parathyroid autotransplantation**, ***n(%)***	28(11.3)	1(3.1)	27(12.5)	0.143^b^
**Wound infection**, ***n(%)***	5(2)	1(3.1)	4(1.9)	0.502^b^
**Hemorrhage**, ***n(%)***	4(1.6)	1(3.1)	3(1.4)	0.427^b^

*p < 0.05, a: Pearson chi-square test, b: Fisher’s exact test

### Comparison of pN0 vs. pN1

#### Patient and tumor characteristics

We performed TT + pCND in 216 patients with PTC. There were 89 patients (41%) without and 127 patients (59%) with CLNM. In the whole group of patients, the mean age was 45.17 ± 12.07; 163 patients (75.5%) were younger than 55 and 53 (24.5%) were 55 or older. There were 40 men (18.5%) and 176 women (81.5%). Classical variant PTC was the predominant histologic subtype, accounting for 57.9% (n:125) of all cases, followed by tall cell variant 31% (n:67) and follicular variant 6.5% (n:14). ETE was noted in 7.9% (n:17) of cases and lymphatic invasion in 76.9% (n:166). Average tumor size was 13.8 mm (median 13 mm, range 2 to 49 mm); 45.4% of tumors were < 10 mm in size and 54.6% were ≥ 10 mm. The majority of tumors was multifocal (69.9%). CND was performed in all patients. On average, 14.54 nodes were found per CND specimen, range 0–49 per specimen, median 13.

#### Clinicopathological associations and RFs predicting CLNM

**Fig. 1:**
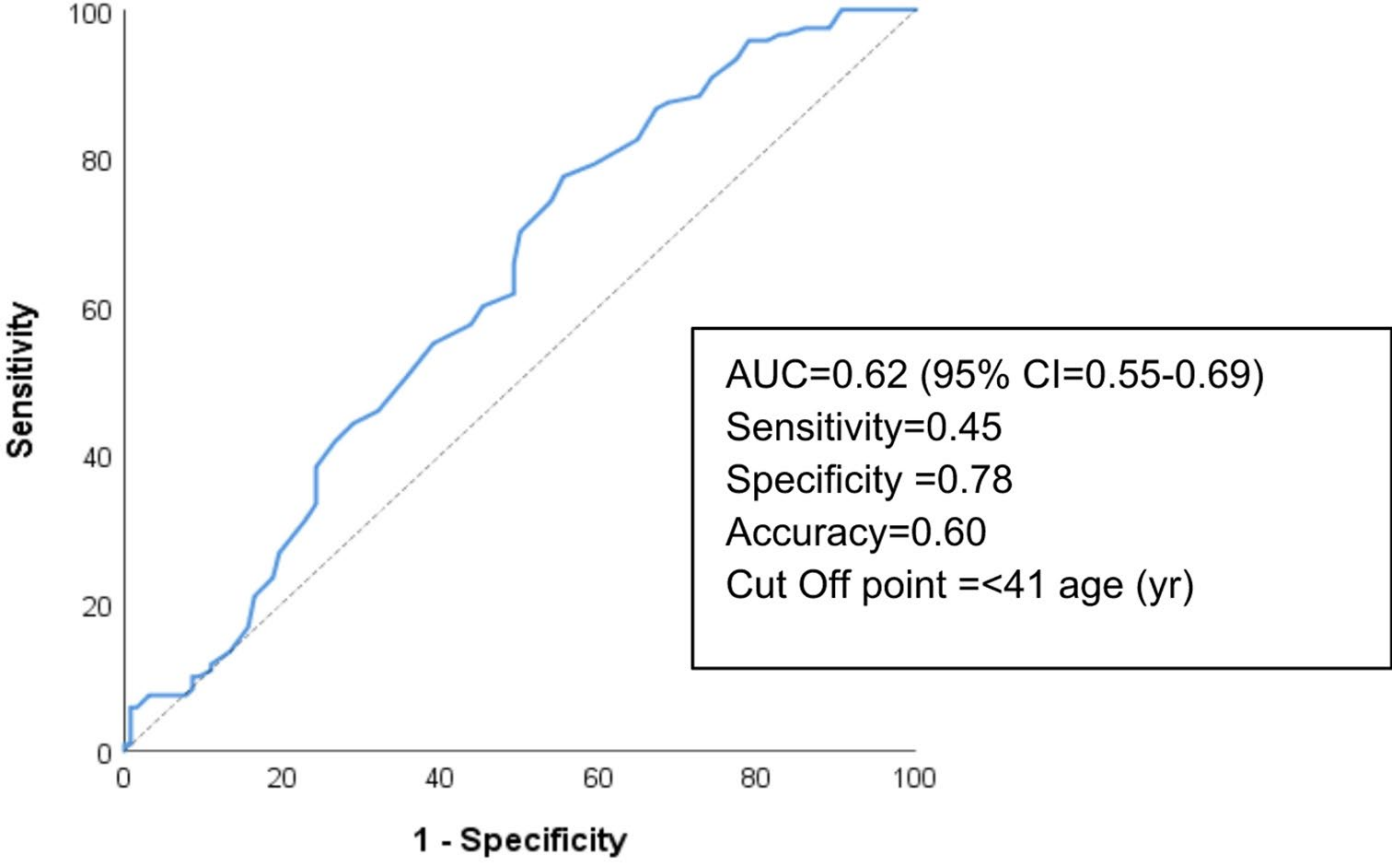
ROC curve

In univariate analysis the mean age of the patients in the pN1 group was lower and compared to the pN0 patient group, the male sex ratio was higher in the pN1 patient group (9% vs. 25%). The most appropriate age limit for determining the presence of pN1 was determined as 41 years (AUC = 0.62). For 41 years of age, sensitivity, specificity and accuracy rates were 45%, 78% and 60%, respectively (Fig. 1). The difference was statistically significant for both age (*p* = 0.032) and sex (*p* = 0.003) (Table 4). In the group of patients with tumors < 1 cm in size we found metastases in 51% of the patients, whereas in the group of patients with tumors ≥ 1 cm metastases were found in almost 65% of patients. Compared to the pN0 patient group, the pN1 patient group had a presence of tumors over 10 mm (40% vs. 75%; *p* = 0.041). It was also found tall cell tumor type (22.5% vs. 37%; *p* = 0.001), ETE (3.4% vs. 11%; *p* = 0.040) and presence of lymphatic invasion (49% vs. 96%; *p* < 0.001) the factors affecting presence of CLNM. The results of the analysis of variables associated with LN involvement are presented in detail in Table 4.

In addition, 14.2% (n:18) of the 127 patients with CLNM were upgraded from the low-risk group to the intermediate-risk group according to the ATA RSS because the number of LNM was ≥ 5. None of these 18 patients had T3 or T4 tumors. When pathological data were analyzed, 58 (26.9%) of 216 patients who underwent pCND received adjuvant treatment only for CLNM, regardless of other poor prognostic factors such as vascular invasion, aggressive histological subtype, ETE and tumor size. Of these 58 patients, 33 patients had macrometastatic LNs although the number of metastatic LNs ≤ 5. The other 25 patients were subjected to dynamic risk classification based on age, postoperative US, postoperative TSH and Tg values as well as histopathological risk classification and adjuvant treatment was decided. From patients with CLNM 51 (23.6%) patients received RAI therapy, due to the combination of RFs such as ETE, aggressive histologic subtype, vascular invasion and LNM. Finally, LN status contributed to the risk classification of the disease and adjuvant treatment decision in 51 (23.6%) of 216 patients who underwent TT + pCND. In patients with CLNM, the micrometastasis rate was 38.5% (n:49), and when the micrometastasis rate was analyzed according to tumor size these rates were 20.4% (n:26), 12.6% (n:16), and 2.4% (n:3) in T1a, T1b, and T2-T3 tumors, respectively.

When we analyzed the clinicopathological RFs that could independently predict CLNM in patients who underwent pCND. Multivariate logistic regression analysis revealed that < 41 years of age [OR = 2.59 (95% CI = 1.23–5.45); *p* = 0.013 ], male sex [OR = 2.26 (95% CI = 1.37–3.71); *p* = 0.001] and the presence of lymphatic invasion [4.09 (95% CI = 2.46–6.80); *p* < 0.001] were found to be independent factors affecting pathological LN involvement (Table 5).

**Table 4:** Variables associated with lymph node involvement

Variables	All (*n* = 216)	pN0 (*n* = 89)	pN1 (*n* = 127)	*P* value
**Age(year)**, ***mean ± SD***	45.17 ± 12.07	47.28 ± 9.79	43.69 ± 13.27	**0.032** ^**a**^ *
**Sex**, ***n(%)***				**0.003** ^**b**^ *
Female	176(81.5)	81(91.0)	95(74.8)	
Male	40(18.5)	8(9)	32(25.2)	
**Tumor size group**, ***n(%)***				**0.041** ^**b**^ *
< 10 mm	98(100)	48(49)	50(51)**	
≥ 10 mm	118(100)	41(44.8)	77(65.2)**	
**Histologic subtype**, ***n(%)***				**0.001** ^**b**^ *
Classic	125(57.9)	51(57.3)	74(58.3)	
Follicular	14(6.5)	9(10.1)	5(3.9)	
Tall cell	67(31)	20(22.5)	47(37)	
Other	10(4.6)	9(10.1)	1(0.8)	
**Multifocal disease**, ***n(%)***	151(69.9)	58(65.2)	93(73.2)	0.204^b^
**Bilaterality**, ***n(%)***	112(51.9)	49(55.1)	63(49.6)	0.430^b^
**Capsular invasion**, ***n(%)***	74(34.3)	32(36)	42(33.1)	0.660^b^
**Extrathyroidal extension**, ***n(%)***	17(7.9)	3(3.4)	14(11)	**0.040** ^**b**^ *
**Vascular invasion**, ***n(%)***	24(11.1)	6(6.7)	18(14.2)	0.087^b^
**Lymphatic invasion**, ***n(%)***	166(76.9)	44(49.4)	122(96.1)	**< 0.001** ^**b**^ *
**TNM stage**, ***n(%)***				**< 0.001** ^**b**^ *
I	184(85.2)	86(96.6)	98(77.2)	
II	32(14.8)	3(3.4)	29(22.8)	
**pT stage**, ***n(%)***				0.276^b^
T1A (< 10 mm)	98(100)	48(49)	50(51)**	
T1B (10–19 mm)	82(100)	28(44.1)	54(65.9)**	
T2 (20–39 mm)	27(100)	10(37)	17(63)**	
T3 (≥ 40 mm)	9(100)	3(33.3)	6(66.7)**	
**ATA Risk Category**, ***n(%)***				**< 0.001** ^**b**^ *
Low	89(41.2)	63(70.8)	26(20.5)	
İntermediate	127(58.8)	26(29.2)	101(79.5)	

*p < 0.05, **a**: Mann‒Whitney U test, **b**: Pearson chi‒square test, **SD**: standard deviation

**Numerical values are given as row percentages

**Table 5:** Independent variables associated with lymph node involvement (multivariate analysis)

Variables	OR(95% CI)	*P* value
**Age***(< 41 year* vs. ≥ 41yr**)	2.59(1.23–5.45)	0.013*
**Sex***(male* vs. female**)	2.26(1.37–3.71)	0.001*
**Lymphatic invasion***(yes* vs. no**)	4.09(2.46–6.80)	0.001*
**Tumor size***(≥ 10 mm* vs. < 10 mm**)	1.02(0.49–2.08)	0.964
**Extrathyroidal extension***(yes* vs. no**)	2.19(0.55–8.69)	0.265
**Histologic subtype**		
* Follicular* vs. Classic**	1.61(0.32–8.15)	0.567
* Tall cell* vs. Classic**	1.53(0.72–3.28)	0.270
* Other* vs. Classic**	0.23(0.02–2.30)	0.212
**Model summary**	Model method = Enter	
	Model, χ²=91.70; *p* < 0.001	
	R^2^_N_ = 0.466	
	AUC = 0.851	

*p < 0.05, **OR**: odds ratio, **CI**: confidence interval, **: reference value

## Discussion

Papillary thyroid cancer is the most commonly diagnosed thyroid malignancy [29]. Increased awareness of thyroid nodular disease, wide availability of US and FNA, and improved accuracy of histopathological examination of surgical samples have been suggested to be reasons for the increased incidence of detection [30]. Fortunately, PTC is usually treatable and has a good prognosis if it is diagnosed early, although it is also accompanied by a high incidence of CLNM [31]. There is no consensus about the need to routinely perform pCND in patients with cN0 PTC. However, the optimal management for those patients is achieved by performing the most appropriate surgery at the time of diagnosis to achieve the best prognosis and minimize the risk to those patients and the need for unnecessary secondary procedures [32]. Therefore, identifying RFs of CLNM could guide surgeons in considering which cN0 PTC patients require pCND.

There are several studies in the literature to identify RFs that may predict CLNM. Xue et al. [33] indicated that RFs for CLNM including age < 45 years, male gender, tumor size of ≥ 1 cm, and ETE predicted CLNM. Age is considered among the most important prognostic factors for TC [34, 35]. Yuan et al. [36] showed that the rate of CLNM was higher in patients < 45 years than that ≥ 45 years. Multivariate analysis showed age < 45 years was independent predictor of CLNM in patients with cN0 PTMC. Kim et al. also observed a trend to an inverse relationship between the incidence of CLNM and age in patients with cN0 PTC [37]. In a recent meta-analysis conducted by Hafez et al. [38] showed age < 45 years, male sex, multifocality, bilaterality, capsular invasion, lymphovascular invasion and ETE are the factors significantly associated with CLNM. Also as reported by Pontius et al. the presence of lymphovascular invasion among patients with PTC is associated with significantly decreased survival [39]. In our study, univariate analysis shows that male gender, young age, the presence of ETE, a primary tumor size ≥ 10 mm, and lymphatic invasion were RFs of CLNM. In multivariate logistic regression analysis we found; <41 years of age [OR = 2.59 (95% CI = 1.23–5.45); *p* = 0.013], male gender [OR = 2.26 (95% CI = 1.37–3.71); *p* = 0.001] and presence of lymphatic invasion [4.09 (95% CI = 2.46–6.80); *p* < 0.001] were found to be independent factors affecting pathologic LN involvement. These results indicate that careful preoperative assessment of LN status must be followed in young and male patients.

In our study, the positive nodes were found in almost 59% of patients of PTC. Statistic analysis showed that the larger the tumor was, the more likely to find CLNM, with the incidence of 51.5, 65.9, 63, and 66.7% in tumor size of; < 1, 1–2, 2–4 cm, and ≥ 4 cm, respectively. These results consist with most reports. Wang et al. [40] also showed a positive correlation between tumor size and the incidence of CLNM, with the incidence of 35.1, 53.9, 47.1, and 100.0% in tumor size of; < 1, 1–2, 2–4 cm, and ≥ 4 cm, respectively. At the same time they found that tumor size was also an independent predictive factor for CLNM in cN0 PTC patients. Roh et al. [41] using multivariate analyses reported that tumor size > 1 cm was an independent factor for ipsilateral CLNM. Park et al. [42] reported that tumor size > 0.7 cm was an independent variable predictive of CLNM in patients with cN0 micro-PTC. However, other studies reported that there was no significant association between the presence of CLNM and tumor size [43, 44]. In this study, multivariate analysis showed that tumor size ≥ 1 cm was not an independent predictor of CLNM in patients with cN0 PTC. However, the incidence of CLNM was much higher in tumors ≥ 1 cm (65.2% versus 44.8%). The difference in size cut-offs in each of those studies might affect the association between the incidence of CLNM and tumor size. Further randomized controlled multicenter study will be helpful to draw the right conclusion.

Both TNM staging system (AJCC) and risk stratification system (ATA) have important prognostic values for TC. The former is used to predict disease-specific mortality, and the latter is mainly used to estimate the LRR. Several studies proposed that pCND did not help LRR and suggested RAI therapy to control the disease [45, 46]. This is in contrast to the findings of Moo et al. [47], who found a trend toward lower recurrence in TT + pCND, which could be due to increased control over local metastases or increased doses of RAI ablation in patients with recognized LNM. Barczynski et al. [26] reported that pCND for more accurate N staging, followed by personalized adjuvant RAI treatment, and significantly improved both 10-year DFS improving from 92.5 to 98.0% and locoregional control, without increasing the risk of permanent morbidity in patients with PTC. Randolph et al. [48] showed that prognosis is negatively impacted if ≥ 5 LNM are identified during neck dissections. In our study, CLNM were found in 59% (127 of 216 cases) of patients with cN0 PTC. Because of the CLNM detected after pCND, 46.7% of patients over 55 years of age were upstaged according to TNM staging. Most of our patients had other RFs that also upgraded them to the ATA intermediate-risk group, however, in 18 patients (14.2%), the CLNM data was the only factor that led to their stratification to the intermediate risk category. From patients with CLNM 51 (23.6%) were recommended to receive the RAI therapy after initial surgery only for CLNM, regardless of other poor prognostic factors. These patients would have been inappropriately down-staged and possibly undertreated if pCND had been omitted. Similar to our results, Zhang et al. [8] reported that the percentage of upstaged patients with cN0 micro-PTC was 16% and Nylen et al. [49] reported that 23% of patients upgraded to intermediate-risk group. They also noted that 4% of these patients were upgraded only because of LN data. Recent studies have suggested that approximately one-third of patients who underwent pCND may have been upstaged [48, 50]. 

Another important reason for pCND is that there is no other reliable method to verify CLNM, whether preoperative or intraoperative US or other methods are far from sufficient sensitivity to verify CLNM [51]. Stulak et al. [52] reported that in a total of 511 patients with PTC, 476 patients were cN0; preoperative US detected CLNM in 10 patients (2.1%), but CLNM were confirmed in 179 patients (32.5%). Preoperative US for the detection of CLNM has high specificity (92%) and positive predictive value (81–92%) but low sensitivity (51–61%) and negative predictive value, especially for central LNs (63–76%) [53, 54]. Therefore, before other reliable authentication methods arise, there is no better way to assess CLNM other than pCND. The detection of CLNM is associated with the administration of higher doses of RAI for postoperative ablation, decreased recurrence in patients undergoing pCND, and the need for reoperation.

Studies have reported higher rates of HPT and RLN palsy with reoperative surgery because of tumor recurrence and local invasion, and LNM is strongly associated with recurrence after TT [55–57]. In the literature, however, the rate of transient HPT has been reported to be between 9.7 and 56.5% after TT + pCND. A permanent HPT has been reported in up to 19.4% of patients after pCND, compared with 0.6–8.1% after simple TT [58]. In this study, results consistent with the literature were obtained. According to the results of a recent meta-analysis published by Yang et al. [59], the TT + pCND group had a significantly higher rate of transient HPT than that of the TT group. This finding was consistent with that of Moo et al. [47], who reported that the number of patients with transient HPT in the TT + pCND group was greater than that in the TT group at the beginning of the study. Some researchers have reached similar conclusions [8, 60–63]. This study revealed that the rates of transient HPT were higher when pCND was performed, but the difference was not statistically significant (30 vs. 18.8; *p* = 0.185). Importantly, the rate of permanent HPT was not different between the 2 groups. We implemented the policy of liberal autografting of parathyroid glands where necessary, which may explain the lower rate of permanent HPT.

The other main complication related to pCND is RLN injury. Roh et al. [62] demonstrated an increased risk of transient RLN palsy after TT + pCND, which could be explained by increased RLN injury caused by dissection of the LNs. However, Wang et al. [64] reported that there were no significant differences in the incidence of other complications, such as transient HPT, permanent HPT, transient RLN palsy and permanent RLN palsy, between the TT and TT + pCND groups. Our data revealed that the rates of transient and permanent RLN palsy were 6.9% and 1.4%, respectively. These results are in accordance with those reported in the literature, which reported rates of transient RLN palsy ranging from 1 to 13% and rates of permanent RLN injury ranging from 0 to 3.6% [65–68]. The addition of CND to TT has been reported to increase neither the risk of transient nor permanent RLN palsy in prophylactic or therapeutic procedures [60, 69–72]. In this study, TT + pCND did not increase the incidence of transient or permanent RLN palsy, but the incidence of transient RLN palsy was lower in the TT + pCND group than in the TT alone group. Due to our approach to signal loss and adverse EMG changes, which we have detailed above, pCND was not applied to any patient when these events was detected. Therefore, we come to the conclusion that the rates of transient RLN palsy were relatively higher in the TT group. In addition, RLN palsy developed due to pCND in only 2 patients in the TT + pCND group in our study. These findings, in line with the literature, led us to advocate that pCND can be safely performed in experienced centers.

The incidence of TC is increasing worldwide. In a large cohort study, an overall 23.3-fold increased risk of TC was observed following the diagnosis of any benign thyroid gland disorder (compared with no diagnosis) [73]. For this reason, especially in our country, which is endemic for thyroid diseases, the treatment approach for PTC gains much more importance. Another striking finding in our study was the high rate of tall cell variants, one of the aggressive subtypes of PTC (31.8%). In the literature, the incidence of tall cell variant is reported to be between 1.3% and 13% [74, 75], and as is well known, tall cell variant has a poorer prognosis with significantly higher rates of LN and distant metastases and lower 5-year DFS [76, 77]. Furthermore, Machens et al. reported that microscopic lymphatic invasion in PTC was associated with LNM and multifocal tumor growth [78]. In our study, the rate of lymphatic invasion was 74% in the whole group and 96% in the group of patients with CLNM.

The first limitation of this study was that it was a retrospective study from a single center, and there might have been selection bias. The second limitation of this study was that we could not evaluate the effects of pCND on the disease-specific survival, locoregional recurrence rates and overall survival of patients with cN0 PTC because the follow-up time was relatively short.

## Conclusion

In conclusion, despite increase in worldwide, PTC has quite high OS rates. From this perspective, prevention of LRR that may develop especially in the central region, secondary surgical complications due to recurrence, DFS and quality of life concepts gain importance. This brings up the determination of the correct surgical strategy during the initial treatment. In our country, where thyroid diseases and thyroid cancer are endemic, considering the frequency of CLNM in cN0 PTC patients, the high rates of aggressive subtypes and lymphatic invasion in our study, we suggest that pCND will be useful in staging, risk assessment, and adjuvant treatment planning of cN0 PTC especially those who carry RFs for CLNM independently of tumor size. We believe that it would be useful to reveal the RFs that will predict CLNM and the contribution of CND to these patients with multicenter studies.

## Data Availability

No datasets were generated or analysed during the current study.
